# Fluoxetine Arrests Growth of the Model Diatom *Phaeodactylum tricornutum* by Increasing Oxidative Stress and Altering Energetic and Lipid Metabolism

**DOI:** 10.3389/fmicb.2020.01803

**Published:** 2020-07-31

**Authors:** Eduardo Feijão, Ricardo Cruz de Carvalho, Irina A. Duarte, Ana Rita Matos, Maria Teresa Cabrita, Sara C. Novais, Marco F. L. Lemos, Isabel Caçador, João Carlos Marques, Patrick Reis-Santos, Vanessa F. Fonseca, Bernardo Duarte

**Affiliations:** ^1^MARE – Marine and Environmental Sciences Centre, Faculdade de Ciências da Universidade de Lisboa, Lisbon, Portugal; ^2^cE3c – Centre for Ecology, Evolution and Environmental Changes, Faculdade de Ciências, Universidade de Lisboa, Lisbon, Portugal; ^3^BioISI – Biosystems and Integrative Sciences Institute, Plant Functional Genomics Group, Departamento de Biologia Vegetal, Faculdade de Ciências da Universidade de Lisboa, Lisbon, Portugal; ^4^Centro de Estudos Geográficos, Instituto de Geografia e Ordenamento do Território, University of Lisbon, Lisbon, Portugal; ^5^MARE – Marine and Environmental Sciences Centre, ESTM, Politécnico de Leiria, Peniche, Portugal; ^6^MARE – Marine and Environmental Sciences Centre, Department of Zoology, Faculty of Sciences and Technology, University of Coimbra, Coimbra, Portugal; ^7^Southern Seas Ecology Laboratories, School of Biological Sciences, The University of Adelaide, Adelaide, SA, Australia; ^8^Departamento de Biologia Animal, Faculdade de Ciências da Universidade de Lisboa, Lisbon, Portugal

**Keywords:** pharmaceuticals, antidepressant, microalgae, ecotoxicity, photobiology, cell energy, biomarkers, fatty acid profile

## Abstract

Pharmaceutical residues impose a new and emerging threat to aquatic environments and its biota. One of the most commonly prescribed pharmaceuticals is the antidepressant fluoxetine, a selective serotonin re-uptake inhibitor that has been frequently detected, in concentrations up to 40 μg L^–1^, in aquatic ecosystems. The present study aims to investigate the ecotoxicity of fluoxetine at environmentally relevant concentrations (0.3, 0.6, 20, 40, and 80 μg L^–1^) on cell energy and lipid metabolism, as well as oxidative stress biomarkers in the model diatom *Phaeodactylum tricornutum*. Exposure to higher concentrations of fluoxetine negatively affected cell density and photosynthesis through a decrease in the active PSII reaction centers. Stress response mechanisms, like β-carotene (β-car) production and antioxidant enzymes [superoxide dismutase (SOD) and ascorbate peroxidase (APX)] up-regulation were triggered, likely as a positive feedback mechanism toward formation of fluoxetine-induced reactive oxygen species. Lipid peroxidation products increased greatly at the highest fluoxetine concentration whereas no variation in the relative amounts of long chain polyunsaturated fatty acids (LC-PUFAs) was observed. However, monogalactosyldiacylglycerol-characteristic fatty acids such as C16:2 and C16:3 increased, suggesting an interaction between light harvesting pigments, lipid environment, and photosynthesis stabilization. Using a canonical multivariate analysis, it was possible to evaluate the efficiency of the application of bio-optical and biochemical techniques as potential fluoxetine exposure biomarkers in *P. tricornutum*. An overall classification efficiency to the different levels of fluoxetine exposure of 61.1 and 88.9% were obtained for bio-optical and fatty acids profiles, respectively, with different resolution degrees highlighting these parameters as potential efficient biomarkers. Additionally, the negative impact of this pharmaceutical molecule on the primary productivity is also evident alongside with an increase in respiratory oxygen consumption. From the ecological point of view, reduction in diatom biomass due to continued exposure to fluoxetine may severely impact estuarine and coastal trophic webs, by both a reduction in oxygen primary productivity and reduced availability of key fatty acids to the dependent heterotrophic upper levels.

## Introduction

Pharmaceuticals and personal care products (PPCPs) are a group of substances that include cosmetics, hormones, antibiotics, antimicrobial agents, and other organic substances (Daughton and Ternes, 1999; Liu and Wong, 2013). According to EUROSTAT (2019), between 2004 and 2018, over 50% of manufactured chemicals were known to be harmful to the ecosystems, of which over 70% had significant environmental impacts. The inefficient removal of these emerging pollutants by wastewater treatment plants (WWTPs) and consequent detection in marine and freshwater environments has raised attention toward the effects of PPCPs on ecosystems (Ebele et al., 2017; Bi et al., 2018).

One of the most commonly prescribed pharmaceuticals is the antidepressant fluoxetine, a selective serotonin re-uptake inhibitor (SSRI) that increases the amount of serotonin at the synaptic cleft (Brooks et al., 2003). Fluoxetine is used to treat various disorders, such as depression, anorexia and nervous bulimia, obsessive compulsive, or panic disorder (El-Bassat et al., 2011), and has a half-life of about 1–4 days in humans and aquatic ecosystems (Lancaster and Gonzalez, 1989; Johnson et al., 2005; Sawyer and Howell, 2011). Given its widespread use, it is not surprising that fluoxetine has been frequently detected in aquatic environments worldwide (Vasskog et al., 2008; Mezzelani et al., 2018; Reis-Santos et al., 2018), in concentrations that reach up to 40 μg L^–1^ (aus der Beek et al., 2016), where it seems to be more persistent than most SSRIs (Johnson et al., 2005; Neuwoehner et al., 2009). Since pharmaceuticals are intended to produce effects at low concentrations in humans (Ebele et al., 2017; Duarte I.A. et al., 2019), and fluoxetine has been reported to be toxic to various groups of organisms (Fent et al., 2006; Corcoran et al., 2010), its presence in marine ecosystems is of emerging concern. In addition, to evaluate the impacts of fluoxetine on invertebrates and vertebrates, it is also paramount that we understand its effects in microorganisms and autotrophs at the base of the marine food webs.

Diatoms are a major group of microalgae, at the base of marine and estuarine food webs, known to produce about 20% of the global primary photosynthetic production and other complex biomolecules such as fatty acids (Domingues et al., 2012). Additionally, diatoms are also one of the major marine carbon sinks and important oxygen-generator agents (Benoiston et al., 2017). Diatoms are able to synthesize essential fatty acids (EFAs), such as the omega 6 (ɷ-6) linoleic acid and the omega 3 (ɷ-3) linolenic acid, which are two major precursors of the long chain polyunsaturated fatty acids (LC-PUFAs), such as eicosapentaenoic acid (EPA) and docosahexaenoic acid (DHA). These play key roles in heart health, immune and inflammatory responses, and neurological tissue structure in animal species (Wiktorowska-Owczarek et al., 2015). Moreover, these LC-PUFA are primarily incorporated through diet by vertebrates that have limited ability to produce them from EFA, and are thus contingent on their production at lower trophic levels (Arts et al., 2001; Parrish, 2009, 2013).

Given its cosmopolitan occurrence (Falkowski, 2002), fully sequenced genome (Bowler et al., 2008), and ability to reflect the early signs of stress, *Phaeodactylum tricornutum* is a renowned species used for ecotoxicological assessments, and is often chosen to evaluate the impact of a myriad of stressors [e.g., temperature (Dodson et al., 2014; Feijão et al., 2018), trace metal pollution (Cabrita et al., 2016, 2017; Matos et al., 2016), nutrient depletion (Abida et al., 2015), or emerging pollutants (Liu et al., 2019)]. Common biochemical responses and physiological mechanisms studied in these studies, include photobiology responses, such as pigment content and electron transport chain (ETC) efficiency variations (Cabrita et al., 2017; Duarte I.A. et al., 2019), oxidative stress responses such as increased antioxidant enzymes activity (Liu et al., 2019) and variations in membrane fatty acids saturation (Abida et al., 2015; Feijão et al., 2018). These physiological responses and metabolic variations in cell composition present great potential as possible biological biomarkers, which according to Van Gestel and Van Brummelen (1996), are any biological entities or responses to a chemical agent, considered at the sub-individual level, measurable or its sub-products, within the organism (Van Gestel and Van Brummelen, 1996).

In this context, the present work aims to evaluate the impacts of fluoxetine exposure on growth, photosynthesis, energetic and fatty acid metabolism, and antioxidant enzymatic defenses on the model diatom *P. tricornutum*, and to evaluate the potential use of these traits as biomarkers of fluoxetine exposure.

## Materials and Methods

### Experimental Setup

Monoclonal cell cultures of *P. tricornutum* Bohlin (Bacillariophyceae) (strain IO 108–01, IPMA), were grown for 4 days at 18 ± 1°C in 250 mL of f/2 medium (Guillard and Ryther, 1962), under constant aeration and a 14/10 h day/night photoperiod. Using a sinusoidal function, the growth chamber was programmed to simulate sunrise and sunset, with light intensity at noon set to simulate a natural light environment (RGB 1:1:1, Maximum PAR 80 μmol photons m^–2^ s^–1^), as previously described by Feijão et al. (2018).

Following the Organization for Economic Cooperation and Development (OECD) guidelines for algae bioassays (OECD, 2011), and the recommended initial cell density guidelines for microalgae cells with similar size to *P. tricornutum*, cell initial concentration was set at 2.7 × 10^5^ cells mL^–1^. Cultures were exposed to a fluoxetine concentration gradient of 0, 0.3, 0.6, 20, 40, and 80 μg L^–1^. The concentrations tested here are within environmentally detected range but also include expected effective concentration ranges and were chosen in order to cover not only a plausible environmental gradient but also typically used ecotoxicological concentrations (Johnson et al., 2007; El-Bassat et al., 2011; Minguez et al., 2014; aus der Beek et al., 2016; Grzesiuk et al., 2016, 2018). Fluoxetine was added to the cultures 48 h after cell inoculation and the exposure period lasted 48 h (OECD, 2011), to ensure that the exposure to fluoxetine was occurring during the exponential growth phase (Cabrita et al., 2017; Feijão et al., 2018). All culture manipulations were performed in a laminar air flow chamber, under standard aseptic conditions.

### Growth Rates and Cell Harvesting

Cell counting of *P. tricornutum* samples under different fluoxetine concentrations was performed in a Neubauer improved counting chamber, coupled with an Olympus BX50 (Tokyo, Japan) inverted microscope, at 400-times magnification. Growth was calculated as the difference between final and initial logarithmic cell densities of *P. tricornutum* divided by the exposure period (Santos-Ballardo et al., 2015), and was expressed as the mean specific growth rate per day. Samples for biochemical analysis were collected after 48 h of exposure to fluoxetine (OECD, 2011). After centrifugation at 4000 × *g* for 15 min at 4°C and supernatant removal, pellets were immediately frozen in liquid nitrogen and stored at −80°C. Three biological replicates for each analysis were collected from a total of 18 experimental units.

### Chlorophyll *a* Pulse Amplitude Modulated Fluorometry

Before sample harvesting, 1 mL of each replicate was used to perform pulse amplitude modulated (PAM) chlorophyll fluorescence measurements using a FluoroPen FP100 (Photo System Instruments, Czechia). Cell density was assessed daily, for comparison purposes, using a non-actinic light (Ft). After cell acclimation for 15 min in the dark, chlorophyll *a* fluorescence induction curves (Kautsky curve) were measured using the OJIP test, according to a pre-programmed standard protocol (Feijão et al., 2018). This test allows to infer on the balance between light incident at the PSII side, the rate of utilization of the (potential) chemical energy, and the rate of heat dissipation (Zhu et al., 2005; Cabrita et al., 2017; Feijão et al., 2018). From this analysis, several photochemical parameters were attained, as shown in Table 1.

**TABLE 1:** Summary of fluorometric analysis parameters and their description.

OJIP-test	
AOECs	Active oxygen-evolving complexes
Area	Corresponds to the oxidized quinone pool size available for reduction and is a function of the area above the Kautsky plot
N	Reaction center turnover rate
S_*M*_	Corresponds to the energy needed to close all reaction centers
P_*G*_	Grouping probability between the two PSII units
ABS/CS	Absorbed energy flux per cross-section
TR/CS	Trapped energy flux per cross-section
ET/CS	Electron transport energy flux per cross-section
DI/CS	Dissipated energy flux per cross-section
RC/CS	Number of available reaction centers per cross-section
TR_0_/DI_0_	Contribution or partial performance due to the light reactions for primary photochemistry
δ_*R*0_/(1-δ_*R*0_)	Contribution of PSI, reducing its end acceptors
ψ_*E*0_/(1-ψ_*E*0_)	Equilibrium constant for the redox reactions between PS II and PS I
RE_0_/RC	Electron transport from PQH_2_ to the reduction of PS I end electron acceptors
RC/ABS	Reaction center II density within the antenna chlorophyll bed of PS II

### Pigment Analysis

Pigments were extracted from sample pellets with 100% cold acetone and maintained in a cold ultra-sound bath for 2 min, to ensure complete disaggregation of the cell material. Extraction proceeded in the dark at −20°C for 24 h, to prevent pigment degradation (Cabrita et al., 2016, 2017; Feijão et al., 2018). Following centrifugation (4000 × *g* for 15 min at 4°C), supernatants were analyzed using a dual beam spectrophotometer. Absorbance spectrums from 350 to 750 nm (0.5 nm steps) were then introduced in the Gauss-Peak Spectra (GPS) fitting library, using SigmaPlot Software. Pigment analysis was employed using the algorithm developed by Küpper et al. (2007), enabling the detection of chlorophyll *a* and *c*, pheophytin *a*, β-carotene, fucoxanthin, diadinoxanthin (DD), and diatoxanthin (DT).

### Fatty Acid Profiles

Following direct trans-esterification of cell pellets, in freshly prepared methanol sulfuric acid (97.5:2.5, v/v), at 70°C for 60 min, fatty acids methyl esters (FAMEs) were recovered using petroleum ether and dried under a N_2_ flow in a dry bath at 30°C (Feijão et al., 2018; Duarte et al., 2019). Hexane resuspended FAMEs were analyzed in a gas chromatograph (Varian 430-GC gas chromatograph) equipped with a hydrogen flame ionization detector set at 300°C. The temperature of the injector was set to 270°C, with a split ratio of 50. The fused-silica capillary column (50 m × 0.25 mm; WCOT Fused Silica, CP-Sil 88 for FAME; Varian) was maintained at a constant nitrogen flow of 2.0 mL min^–1^ and the oven set at 190°C. Fatty acids identification was performed by comparison of retention times with standards (Sigma-Aldrich), and chromatograms analyzed by the peak surface method, using the Galaxy software. The internal standard used was the pentadecanoic acid (C15:0). The double bond index (DBI), a typical indicator of membrane saturation levels (Feijão et al., 2018) was calculated as follows:

DBI=2×(%monoenes+2×%dienes+3×

$$\frac{\%\text{trienes}+4\times \%\text{tetraenes}+5\times \%\text{pentaenes})}{100}$$

### Lipid Peroxidation

Lipid peroxidation products were determined according to Heath and Packer (1968). Cell pellets were homogenized in 1.5 mL of cold 10% trichloroacetic acid (TCA) and placed in a cold ultra-sound bath for 1 min and subsequently at 100°C for 30 min. After stopping the reaction in ice extracts were centrifuged at 15,000 × *g* for 10 min at 4°C. Subsequently, 1 mL of the supernatant was collected and added to 1 mL of 0.4% thiobarbituric acid (TBA) and extracted at 95°C for 30 min. Samples were cooled on ice and centrifuged as before. Absorbance at 532 and 600 nm was detected by spectrophotometry. The concentration of malondialdehyde (MDA) was calculated using the molar extinction coefficient, 155 mM^–1^ cm^–1^.

### Antioxidant Enzyme Assays

The soluble protein fraction was extracted from cell pellets in 1 mL of 50 mM sodium phosphate buffer (pH 7.6) with 0.1 mM Na-EDTA, followed by sonication for 1 min. Samples were centrifuged (10,000 × *g* for 10 min at 4°C) and the supernatant was collected. Protein content was determined according to Bradford (1976). Catalase (CAT) activity was determined by H_2_O_2_ consumption and consequent decrease at a 240 nm absorbance (ε = 39.4 mM^–1^ cm^–1^) (Teranishi et al., 1974) in a reaction medium containing 50 mM of sodium phosphate buffer (pH 7.6), 0.1 mM of Na-EDTA, 100 mM of H_2_O_2_ and 100 μL of protein extract.

Ascorbate peroxidase (APX) was assessed according to the protocol of Tiryakioglu et al. (2006), monitoring a decrease in absorbance at 290 nm (ε = 2.8 mM^–1^ cm^–1^) in a reaction mixture with 50 mM of sodium phosphate buffer (pH 7.0), 5 μM of H_2_O_2_, 0.25 μM of L-ascorbate and 100 μL of the extract. The SOD activity was evaluated according to Marklund and Marklund (1974) by monitoring the reduction of pyrogallol at 325 nm in a reaction mixture composed by 10 μL of extract, 50 mM of sodium phosphate buffer (pH 7.0), 0.24 mM of pyrogallol and Milli-Q water. Control assays were done in the absence of substrate to evaluate the autoxidation of the substrates. All enzyme extractions and consequent assays were performed at 4 and 25°C, respectively.

### Energy Balance

After cell pellet homogenization using 1 mL of Milli-Q water and brief ultrasonication, to further evaluate lipid, carbohydrate and protein contents and electron transport system (ETS) activity, aliquots were collected from each sample for each analysis. Milli-Q water was used as reaction blank in all assays. The spectrophotometric measurements were performed in triplicates, at 25°C, in a synergy H1 Hybrid Multi-Mode microplate reader (Biotek Instrument, VT, United States).

#### Available Energy

To determine the energy available, the total content of proteins, carbohydrates and lipids were transformed into energetic equivalents, by using the respective energy of combustion (17,500 mJ mg carbohydrates^–1^, 24,000 mJ mg protein^–1^, and 39,500 mJ mg lipid^–1^) (Gnaiger, 1983).

Total lipids, proteins and carbohydrates extraction and further quantification were performed according to De Coen and Janssen (1997, 2003), with minor modifications (Aderemi et al., 2018). Briefly, cell pellets were resuspended in 50 mM sodium phosphate buffer (pH 7) containing 1 mM phenylmethylsulfonyl fluoride (PMSF). Cells were disrupted with 0.42–0.6 mm glass beads (Sigma-Aldrich) for 15 min at 6.5 ms^–1^ in a bead beater (FastPrep-24, MP Biomedicals). The cell extract was centrifuged at 10,000 *g* for 20 min at 4°C. The supernatant was stored at −80°C until analysis. Total protein content in the samples was determined using Bradford’s method (Bradford, 1976). Total lipids were extracted following the method of Bligh and Dyer (1959), by adding to 150 μL of sample, 250 μL of chloroform (spectrophotometric grade, Sigma-Aldrich), 250 μL of methanol (spectrophotometric grade, Sigma-Aldrich) and 125 μL Milli-Q water were added. After centrifugation at 1000 *g* for 5 min, the organic phase and interphase were removed and 500 μL of H_2_SO_4_ was added to 100 μL of lipid extract and charred for 15 min at 200°C. After cooling down to 20°C, 1.5 mL of deionized water was added, and total lipid content was determined by measuring the absorbance at 375 nm and compared to a calibration using tripalmitin as standard. total carbohydrate content was determined by adding 50 μL of 15% TCA was added to the 150 μL of sample and held at −20°C for 10 min. After centrifugation at 1000 *g* for 10 min, total carbohydrate content of the supernatant fraction was quantified by adding 50 μL of 5% (v/v) phenol and 200 μL of 18 M H_2_SO_4_ to 50 μL extract (De Coen and Janssen, 1997). After 30 min of incubation at 20°C, the absorbance was measured at 492 nm and compared to a calibration using glucose as standard.

#### Energy Consumption

As reported by King and Packard (1975), the mitochondrial ETS activity has been proposed as a valid alternative to whole organism respiration rates, since it is directly linked to cellular oxygen consumption and metabolism. Therefore, ETS was measured according to King and Packard (1975) with major modifications described previously (Aderemi et al., 2018). Briefly, to 30 μL of sample or blank, 20 μL of homogenizing buffer [0.3 M Tris, 15% (w/v) polyvinyl pyrrolidone (PVP), 459 μM MgSO_4_, 1.5 ml Triton X-100, pH 8.5], and 100 μL of buffered substrate solution [reduced nicotinamide adenine dinucleotide (NADH) (1.79 mM) and reduced nicotinamide adenine dinucleotide phosphate (NADPH) (280 μM) in 0.13 M Tris, 0.3% (w/v) Triton X-100, pH 8.5] were added. The reaction was started with the addition of 50 μL of 8 mM p-iodonitrotetrazolium (INT) and the change in absorbance measured at 490 nm over a 3 min period at 20°C. The amount of formazan formed was calculated by using extinction coefficient, ε = 15,900 mM^–1^ cm^–1^.

Based on the theoretical stoichiometrical relationship that for each 2 μmol of INT-formazan formed, 1 μmol of O_2_ was consumed in the ETS, the cellular energy consumption (Ec) was determined using the ETS results.

The quantified oxygen consumption was transformed using the specific oxyenthalpic equivalents (energy equivalents obtained from the consumption of oxygen) for an average lipid, protein, and carbohydrate mixture of 480 kJ mol O_2_^–1^ into energetic equivalents (Gnaiger, 1983).

#### Cellular Energy Allocation

The cellular energy allocation (CEA) values, standardized to 10^6^ cells, were determined based on measurements of lipid, carbohydrate, and protein content and ETS activity for each sample, as follows (Verslycke et al., 2004):

$$\text{CEA}=\frac{\text{Ea}}{\text{Ec}}$$

Where:

Ea(availableenergy)=carbohydrate+lipid+protein

$$\left(mJ/10^{6}\mathrm{cells}\right)$$

$$\text{Ec}\left(\mathrm{energy}\mathrm{consumption}\right)=\mathrm{ETS}\mathrm{activity}\left(\mathrm{mJ}/10^{6}\mathrm{cells}/h\right)$$

### Statistical Analysis

For each variable considered in this study (i.e., growth, photobiological, and biochemical variables), differences among fluoxetine concentrations were evaluated through non-parametric Kruskal–Wallis tests, due to lack of normality and homogeneity of variances. Spearman correlation tests were applied to assess the relationship between the exogenous dose applied and the photochemical and biochemical variables. Both Kruskal–Wallis and Spearman tests were performed using Statistica Software (StataSoft). In order to test for changes in whole photochemical metabolism and fatty acid profiles, a multivariate approach was considered (Duarte et al., 2017a, b). Canonical analysis of principle coordinates (CAP), using Euclidean distances, was used to visualize differences in multivariate space regarding fatty acid relative composition and studied photochemical variables, as well as to determine how accurately samples could be allocated to different treatment groups. This multivariate approach is insensitive to heterogeneous data and frequently used to compare different sample groups using the intrinsic characteristics of each group (metabolic characteristics) (Cabrita et al., 2017; Duarte et al., 2017b, 2018). Multivariate statistical analyses were conducted using Primer 6 software (Clarke and Gorley, 2006). Statistical significance was considered when *p* < 0.05.

## Results

### Cell Growth Rates

This experiment lasted for 96 and 48 h after cell inoculation, which corresponds to a lag phase or adaptation period, cells were exposed to different concentrations of fluoxetine as they entered the exponential phase. Cells were analyzed during this phase to minimize the effects often caused by nutrient depletion and culture aging, resulting from long experimental periods (Cabrita et al., 2016; Feijão et al., 2018). Although some replicates seem to grow slower than the control samples during the first 48 h, it is important to notice that each replicate started with the same amount of cells and small variations in cell density during this lag phase can be related to minor variations in aeration, manual sampling or counting and even the microenvironment inside each flask. However, these small differences tend to get smaller as the culture progresses.

**FIGURE 1:**
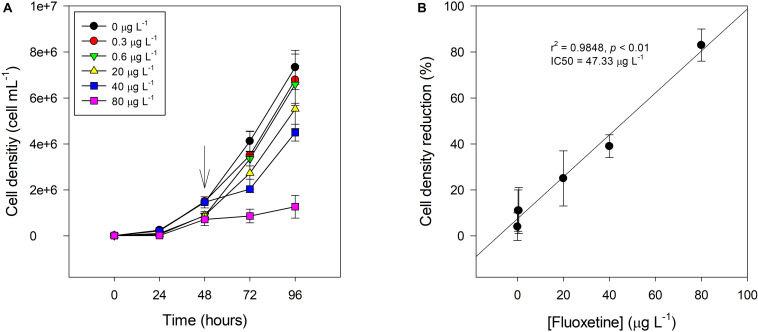
Cell density **(A)** and relative cell density reduction toward the control **(B)** of *Phaeodactylum tricornutum* following a 48 h exposure to different fluoxetine concentrations. *X*-axis in **(A)** includes the whole experiment duration from algae inoculation (0 h) to fluoxetine exposure (48 h) and cell harvest (96 h) and storage. Arrow points to the moment of initial fluoxetine exposure of *P. tricornutum* cells. Average ± standard deviation.

Cultures of *P. tricornutum* cells exposed to increasing concentrations of fluoxetine presented a lower cell density after 48 h exposure when compared to control (Figure 1A). The effect of fluoxetine exposure on cell density was exacerbated at higher concentrations (40 and 80 μg L^–1^). Cultures exposed to 40 and 80 μg L^–1^ of fluoxetine presented an inhibitory percentage of 39 ± 5 and 83 ± 7%, respectively, thus the concentration that inhibits half the maximum growth (IC_50_) was approximately 47.3 μg L^–1^ (Figure 1B). Overall, a high positive correlation between growth inhibition presented as relative cell density reduction and increasing fluoxetine concentration (*r*^2^ = 0.98, *p* < 0.05) was found.

### Diatom Photochemistry

**FIGURE 2:**
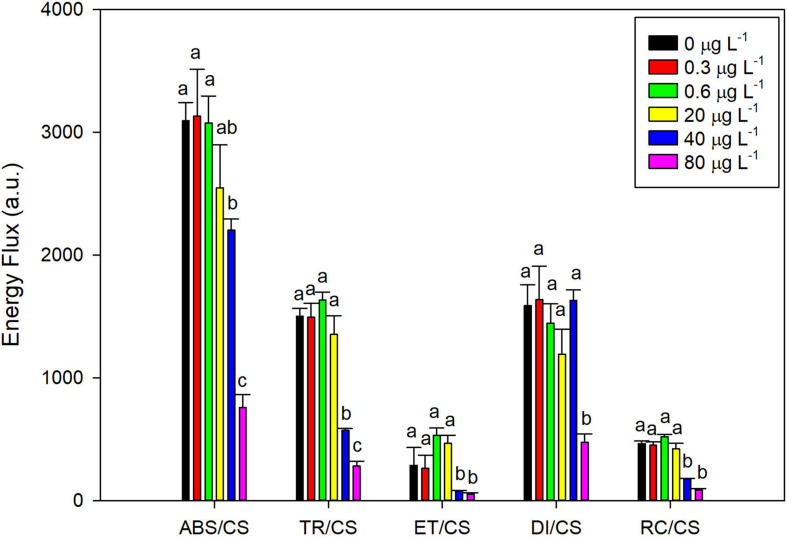
*Phaeodactylum tricornutum* phenomenological energy fluxes: absorbed (ABS/CS), trapped (TR/CS), transported (ET/CS), dissipated (DI/CS) and number of available reaction centers per cross section (RC/CS), following a 48 h exposure to different fluoxetine concentrations. Average ± standard deviation, *N* = 3, different letters indicate significant differences at *p* < 0.05.

Analyzing the four main energy fluxes that represent the overall photochemical process from light harvesting electronic transport, there was a striking decrease in all parameters on *P. tricornutum* cells exposed to the highest levels of fluoxetine (40 and 80 μg L^–1^) (Figure 2). At these concentrations, a decrease in energy absorption, trapping, transport, and dissipation was observed when compared to control samples. Moreover, a dose-response effect in the absorbed and trapped energy fluxes was evident (*r*^2^ = −0.77 and *r*^2^ = −0.74, respectively at *p* < 0.05). The number of reaction centers per culture cross section also showed a marked decreased (*r*^2^ = −0.75, *p* < 0.05).

**FIGURE 3:**
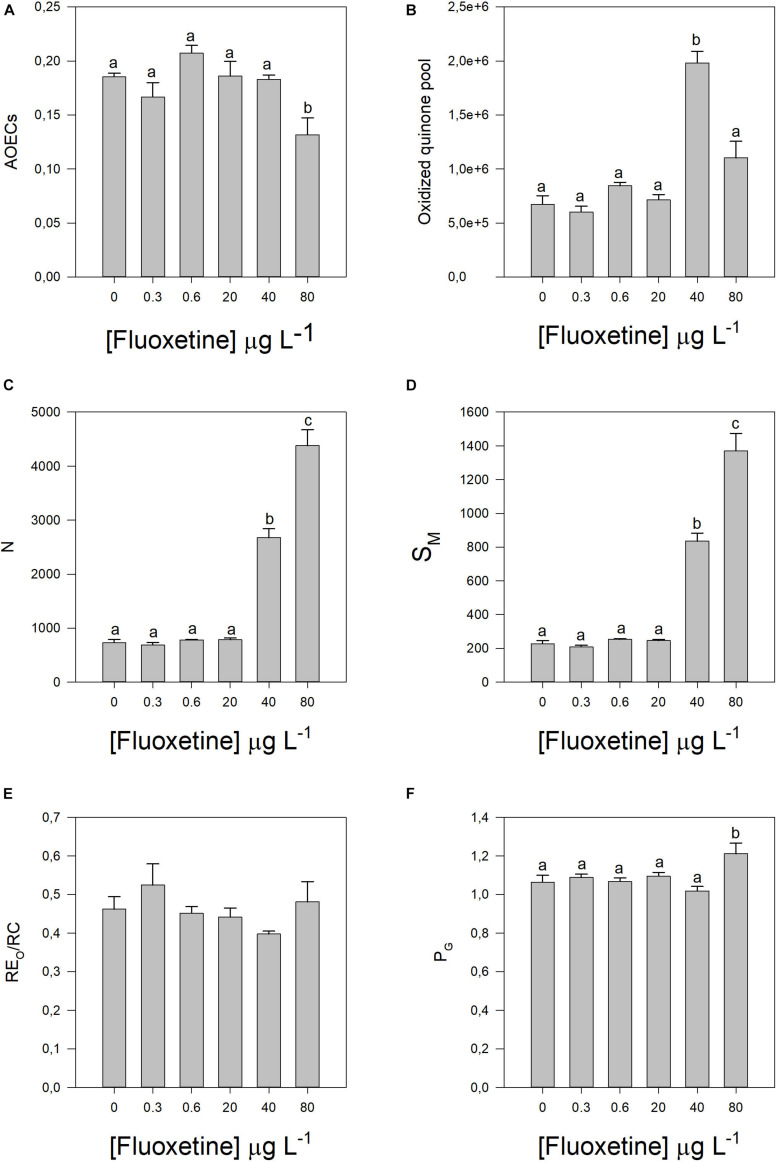
Parameters derived from OJIP transient curves in *Phaeodactylum tricornutum* cells following a 48 h exposure to different fluoxetine concentrations. **(A)** Active oxygen-evolving complexes (AOECs). **(B)** Oxidized quinone pool (area). **(C)** Turnover number of Q_*A*_ (N). **(D)** Relative pool size of plastoquinone (S_*M*_). **(E)** Electron transport from PQH_2_ to the reduction of PS I end electron acceptors (RE_0_/RC). **(F)** and Grouping probability (P_*G*_). Average ± standard deviation, *N* = 3, different letters indicate significant differences at *p* < 0.05.

**FIGURE 4:**
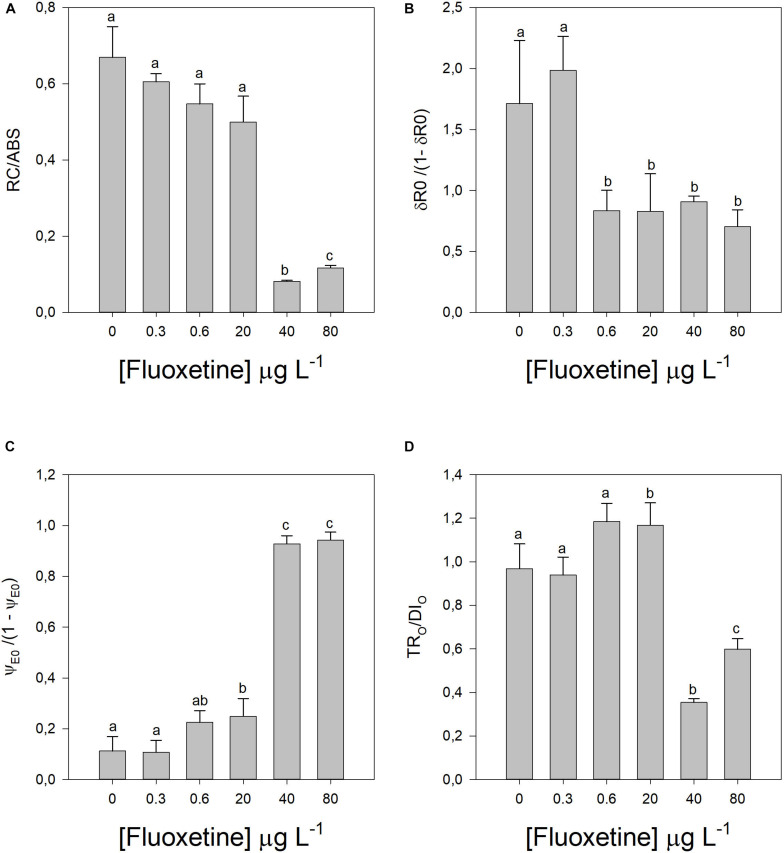
Parameters derived from OJIP transient curves in *Phaeodactylum tricornutum* cells following a 48 h exposure to different fluoxetine concentrations. **(A)** Reaction center II density within the antenna chlorophyll bed of PS II (RC/ABS). **(B)** Contribution of PSI, reducing its end acceptors [δR0/(1–δR0)]. **(C)** Equilibrium constant for the redox reactions between PS II and PS I [ψE0/(1-ψE0)]. **(D)** Contribution or partial performance due to the light reactions for primary photochemistry (TR_0_/DI_0_). Average ± standard deviation, *N* = 3, different letters indicate significant differences at *p* < 0.05.

These energy transduction variations can be caused by changes in different components of the photochemical apparatus (Figures 3, 4). Increasing fluoxetine concentrations, decreased active oxygen-evolving complexes (AOECs), alongside with an increase in the oxidized quinone pool size (Area, *r*^2^ = 0.69, *p* < 0.05), Q_*A*_ turnover (N, *r*^2^ = 0.81, *p* < 0.05) and the relative plastoquinone (PQ) pool size (S_*M*_, *r*^2^ = 0.81, *p* < 0.05) (Figures 3A–D). The electron transport from PQH_2_ to the reduction of PS I end electron acceptors (RE_0_/RC) did not show any significant variation (Figure 3E). Disconnectivity between PS II antennae (PG) increased (Figure 3F). The number of Q_*A*_ reducing RCs *per* PSII antenna drastically decreased with increasing fluoxetine concentration (*r*^2^ = −0.81, *p* < 0.05) (Figure 4A).

The involvement of the PSI on its end-acceptors reduction significantly decreased in cells exposed to concentrations of fluoxetine above 0.6 μg L^–1^ (δR0/(1−δR0), *r*^2^ = −0.58, *p* < 0.05), however this only produced significant increases in the equilibrium constant of the redox reactions between PS II and PS I, at fluoxetine concentrations above 20 μg L^–1^ (ψE0/(1−ψE0), *r*^2^ = 0.86, *p* < 0.05; Figures 4B,C). On the other hand, the contribution or partial performance due to light reactions for primary photochemistry decreased with increasing fluoxetine (TR_*O*_/DI_*O*_, *r*^2^ = −0.50, *p* < 0.05).

### Effects on Diatom Pigment Composition

**FIGURE 5:**
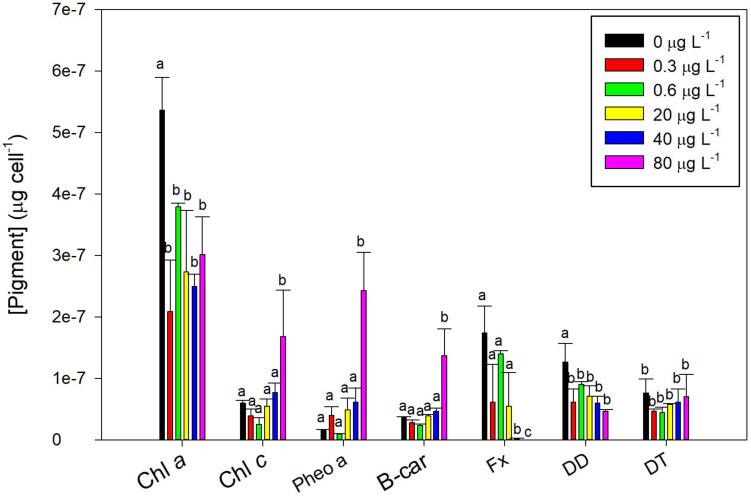
*Phaeodactylum tricornutum* pigment profile following a 48 h exposure to different fluoxetine concentrations. Pigments include chlorophyll *a* (Chl *a*), chlorophyll *c* (Chl *c*), pheophytin *a* (Pheo *a*), β-carotene (B-car), fucoxanthin (Fx), diadinoxanthin (DD), and diatoxanthin (DT) content. Average ± standard deviation, *N* = 3, different letters indicate significant differences at *p* < 0.05.

Fluoxetine exposure lead to significant changes in the fucoxanthin-chlorophyll protein (FCP) related pigments of *P. tricornutum* (Figure 5). All the concentrations tested induced a decrease in the most abundant photosynthetic pigment of *P. tricornutum*, chl *a*, but only the highest concentration significantly enhanced chl *c* (*r*^2^ = 0.57, *p* < 0.05) and decreased fucoxanthin (Fx, *r*^2^ = −0.58, *p* < 0.05) concentrations. Chlorophyll *a* degradation product, pheophytin *a*, showed a positive dose-response relationship (*r*^2^ = 0.61, *p* < 0.05) and drastically increased in cells exposed to highest fluoxetine concentration (80 μg L^–1^).

The carotenoids involved in the diatom photoprotection and antioxidant mechanisms are the β-car, DD, and DT. The β-carotene levels only showed significant increases under exposure to highest fluoxetine concentrations (*r*^2^ = 0.74, *p* < 0.05). On the other hand, diadinoxanthin was reduced with increasing concentrations (*r*^2^ = −0.55, *p* < 0.05), whilst diatoxanthin showed no significant variations.

### Lipid Peroxidation Products and Antioxidant Enzymatic Activity

**FIGURE 6:**
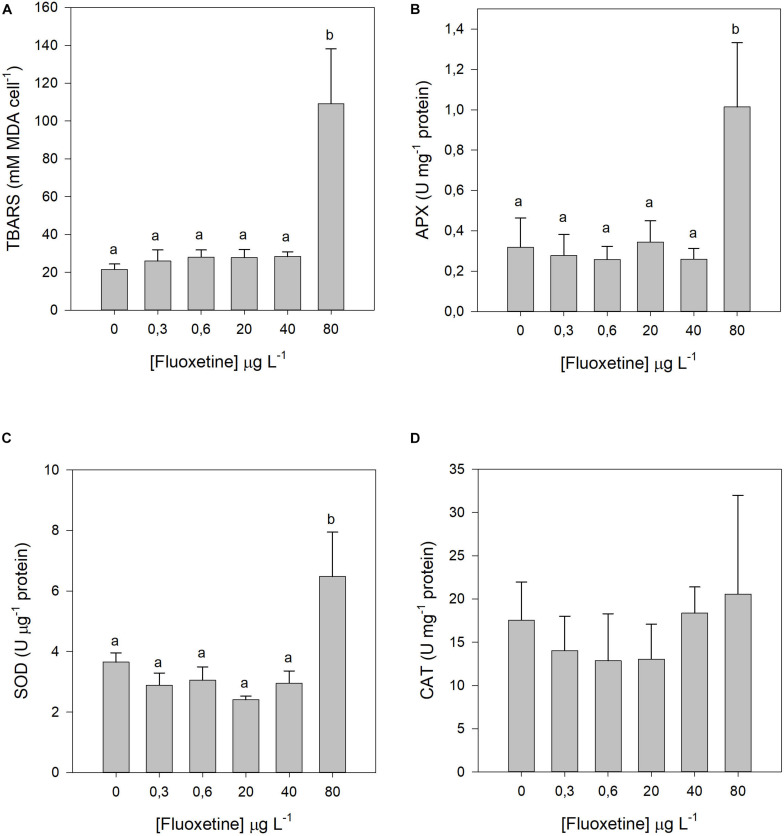
Lipid peroxidation products and antioxidant enzymatic activities of *Phaeodactylum tricornutum* cells following a 48 h exposure to different fluoxetine concentrations. **(A)** Lipid peroxidation products (TBARS). **(B)** Ascorbate peroxidase (APX). **(C)** Superoxide dismutase (SOD). **(D)** Catalase (CAT). Average ± standard deviation, *N* = 3, different letters indicate significant differences at *p* < 0.05.

A significant rise in lipid peroxidation products was only observed in *P. tricornutum* cells exposed to the highest fluoxetine concentration (Figure 6A). Among the antioxidant enzymes tested, SOD and APX followed a similar trend to lipid peroxidation, whereas CAT activity did not respond to any of the tested fluoxetine concentrations (Figures 6B–D). No dose-response relationships were observed for any of the oxidative stress biomarkers.

### Fatty Acid Profile

**FIGURE 7:**
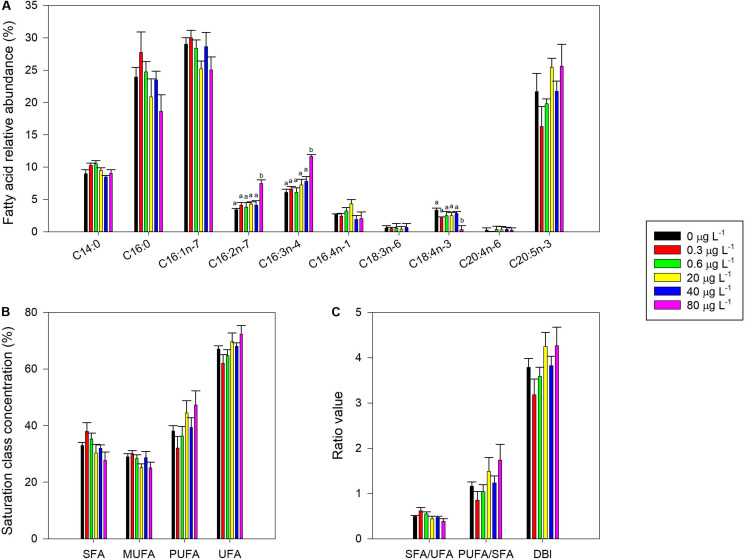
Fatty acid profile of *Phaeodactylum tricornutum*, following a 48 h exposure to different concentrations of fluoxetine. **(A)** Individual fatty acids (FA) abundance. **(B)** Major FA saturation classes relative abundance [saturated fatty acids (SFA), monounsaturated fatty acids (MUFA), polyunsaturated fatty acids (PUFA) and unsaturated fatty acids (UFA) relative abundance]. **(C)** FA ratios [saturated to unsaturated fatty acids ratio (SFA/UFA), polyunsaturated to saturated fatty acids ratio (PUFA/SFA) and double bound index (DBI)] are presented. Average ± standard deviation, *N* = 3, different letters indicate significant differences at *p* < 0.05.

The most abundant fatty acids identified in *P. tricornutum* cells were the saturated myristic (C14:0) and palmitic (C16:0) acids, the monounsaturated palmitoleic acid (C16:1), the di-unsaturated hexadecadienoic acid (C16:2n-7), the tri-unsaturated hexadecatrienoic acid (C16:3), and the LC-PUFA EPA (C20:5). Smaller amounts of hexadecatetraenoic acid (C16:4), γ-linolenic acid (C18:3), stearidonic acid (C18:4), and arachidonic acid (C20:4) were also detected (Figure 7). Although no significant effects were found under pair-wise comparisons, some fatty acids concentrations showed a dose-response relationship. In particular, palmitic, palmitoleic and stearidonic acids showed an inverse relationship with fluoxetine concentrations (*r*^2^ = −0.68, *r*^2^ = −0.54, and *r*^2^ = −0.48, respectively at *p* < 0.05). On the other hand, hexadecadienoic, hexadecatrienoic and EPA acid increased with exposure to increased levels of fluoxetine (*r*^2^ = 0.66, *r*^2^ = 0.81, and *r*^2^ = 0.58, respectively at *p* < 0.05). No significant differences in fatty acid derived parameters (i.e., DBI, saturation classes, and ratios) were found (Figures 7B,C). Nevertheless, some of these parameters showed significant (*p* < 0.05) negative correlations with exogenous fluoxetine concentrations (*r*^2^_*SFA*_ = −0.69, *r*^2^_*MUFA*_ = −0.54, *r*^2^_*SFA/UFA*_ = −0.69), while others showed the inverse trend (*r*^2^_*PUFA*_ = 0.69, *r*^2^_*UFA*_ = 0.69, *r*^2^_*PUFA/SFA*_ = 0.66, *r*^2^_*DBI*_ = 0.60). Total fatty acid content was not affected by the fluoxetine concentrations tested (not shown).

### Energy Balance

**FIGURE 8:**
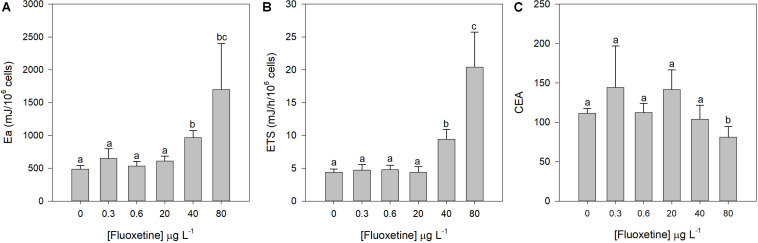
Available energy (Ea, **A**), electron transport system (ETS) at the mitochondrial level **(B)**, and cellular energy allocation (CEA, **C**) changes in *Phaeodactylum tricornutum* cells exposed to fluoxetine for 48 h. Average ± standard deviation, *N* = 3, different letters indicate significant differences at *p* < 0.05.

Exposure of *P. tricornutum* cells to increasing fluoxetine concentrations caused a rise in available energy, mostly due to a lipid and protein content increase as opposed to a carbohydrate content decrease (data not shown); and energy consumption at concentrations of 40 and 80 μg L^–1^ (Figures 8A,B). CEA only decreased significantly at the highest fluoxetine concentration (Figure 8C). CEA was not correlated with fluoxetine concentration (*r*^2^_*CEA*_ = −0.26), however available energy and energy consumption evidenced significant positive correlations (*r*^2^_*Ea*_ = 0.70 and *r*^2^_*ETS*_ = 0.83, *p* < 0.05) with increasing fluoxetine concentrations.

### Multivariate Classification

**FIGURE 9:**
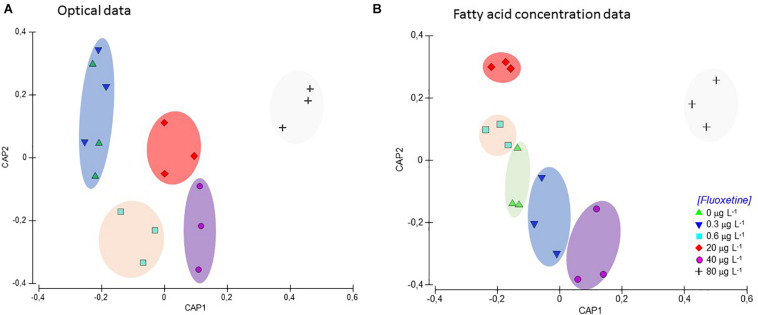
Plots of canonical analysis of principle coordinates (CAP) based on optical data (values for each point of the Kautsky curve), **(A)** and fatty acid profiles (relative abundance of each fatty acid detected). **(B)** of *Phaeodactylum tricornutum* exposed to different fluoxetine concentrations for 48 h. Ellipses group samples with lower statistical distance.

The multivariate CAP analysis using photochemical data and fatty acid profiles produced similar high classifications to groups of origin (Figure 9). CAP analysis based on the Kautsky plot raw fluorescence data was calculated using all the fluorescence data attained during the OJIP-test. This effectively separated five groups in the multivariate canonical space (overall correct classification to treatment of origin = 61.1%). Specifically, control samples and samples exposed to the lowest fluoxetine concentration (0.3 μg L^–1^) were grouped together while the other four concentrations were clearly separated (Figure 9A). On the other hand, the CAP analysis based on fatty acid concentration (constructed having as input the relative concentration of each fatty acid) successfully separated all the different test groups, with an overall classification accuracy of 88.9% (Figure 9B).

## Discussion

Higher concentrations of fluoxetine clearly reduced the cell density of *P. tricornutum*. Growth inhibition or relative cell density reduction reached its higher value (approximately 83%) at 80 μg L^–1^, and an IC_50_ of 47.3 μg L^–1^ was observed, which is in agreement with results previously reported for the marine diatom *Skeletonema marinoi* and for the freshwater microalgae *Raphidocelis subcapitata* (IC_50_ of 44.99 μg L^–1^ and 48.2 μg L^–1^, respectively) (Johnson et al., 2007; Minguez et al., 2018). Bi et al. (2018) studied the impact of fluoxetine exposure on green algae, using a wide range of concentrations (0–1280 μg L^–1^), and concluded that fluoxetine was very stable in the water, leading to severe inhibitory effects, as it could also be observed in the present work. Considering reported environmental fluoxetine concentrations in estuarine waters, higher than 40 μg L^–1^ (aus der Beek et al., 2016), the currently identified IC_50_ value suggests that planktonic diatom biomass could be significantly affected during exposure events.

Variations in *P. tricornutum* growth due to stressors such as metal exposure and temperature are often coupled to photosynthesis impairment, pigment degradation and changes in fatty acids contents and composition (Kudo et al., 2000; Cabrita et al., 2017; Feijão et al., 2018; Duarte et al., 2019). Pigment analysis revealed that *P. tricornutum* cells exposed to fluoxetine suffered a decrease in the most abundant components of the FCP complex, chl *a* and Fx. These changes are probably due to chlorophyll degradation, since a concomitant increase in pheophytin *a* concentration is observed, and could explain the severe reduction in absorption, transport and dissipation of energy fluxes. Furthermore, the density of RCs also declined (RC/CS). Negative impacts in pigments concentration, namely chlorophyll *a* have been reported in *P. tricornutum* when cells are exposed to stresses such as pesticides (Ibrahim, 1983), trace metals (Cid et al., 1995, 1997; Kudo et al., 2000; Cabrita et al., 2017), high light (Janssen et al., 2001), and nutrient deprivation (Zhao et al., 2014). Exposure to other SSRIs and fluoxetine has also been reported to cause decreases in abundance of chlorophyll *a* in freshwater plankton colonizing quartz rock substrates under microcosm conditions (Yang et al., 2019) and in algal biofilms (Richmond et al., 2019).

Exposure to the highest concentrations of fluoxetine seemed to affect more prominently the donor side of the PSII, as observed by the decrease in the fraction of active OECs. On the other hand, the ETC was not severely affected, as reflected by the increased oxidized quinone pool (area), the increased PQ pool size (S_*M*_) and Q_*A*_ turnover. Although the contribution or partial performance due to light reactions for primary photochemistry decreased (lower TR_0_/DI_0_), directly connected to PSII activity, the PSI also decreased its contribution in reducing its ends acceptors. The outcome of both decreases induced a shift in the redox equilibrium constant toward PSII, since the decrease in photosystem activity was more severe in PSI. One of the major impacts of decreased PSI activity is an inefficient recycling of NADP to NADPH (through the ferredoxin system), which will affect several other photosynthetic processes, such as carbon fixation within the Calvin cycle. Additionally, one of the major sources of superoxide radical (O_2_^⋅–^) production is the primary electron acceptor of PS I (Scarpeci et al., 2008; Jahns and Holzwarth, 2012; Ivanov et al., 2018). Hence, lower PSI activity would contribute to a significant accumulation of electron potential, a major source of O_2_^⋅–^ radicals. Another important source of this deleterious anion, is Q_*A*_ which when restricted and over reduced can promote electron leakage to O_2_ (Cleland and Grace, 1999; Takagi et al., 2016; Ivanov et al., 2018). The potential increased ROS production, namely O_2_^⋅–^, is also supported by a rise of lipid peroxidation, which can lead to singlet oxygen (^1^O_2_) production (Pospíšil, 2016) and activation of photoprotection mechanisms such as β-car, a powerful quencher of ^1^O_2_ (Telfer et al., 2002; Telfer, 2014). On the other hand, the high activity of SOD producing H_2_O_2_ (by superoxide anion dismutation), and its consequent metabolization by APX and CAT, produces inevitably hydroxyl radicals (Takagi et al., 2016), that without the iron-based Fenton reactions will be free in the cell, and prone to generate lipid hydroperoxides.

The observed decrease of the Fx and DD synthesis, suggests a possible blockage of the biosynthetic pathway at the β-carotene level, since this molecule is one of their first precursors in this pathway (Kuczynska et al., 2015). Impaired carotenoid synthesis could also be linked to the observed increase in lipid peroxidation since DD-cycle pigments have been reported to prevent lipid peroxidation and scavenge ^1^O_2_ (Lavaud et al., 2004; Kuczynska et al., 2015). In fact, in our study, the diadinoxanthin content showed a significant dose-effect relationship, being reduced as fluoxetine concentration increased. The cells show no sign of photoinhibition (low dissipation energy flux), but rather a severe decrease in the absorbed, trapped, and transported energy fluxes. In terms of metabolic needs, diatom cells exposed to high fluoxetine concentrations apparently need more carotenoid-based ROS quenchers and less pigments like DD and DT which are the most important short-term photoprotection pigments under high light conditions (Grouneva et al., 2009; Lepetit et al., 2010; Jahns and Holzwarth, 2012).

The intense electron flux associated to ATP production by oxidative phosphorylation in the mitochondrial ETC can lead to ROS accumulation, due to increased formation and/or inefficient scavenging of these deleterious molecules under stress conditions (Aderemi et al., 2018). One of the key ROS production sites is the NADH dehydrogenase at complex I (Lesser, 2006) where O_2_^⋅–^ synthesis can occur, highly potentiated by changes in the redox state of the respiratory chain (Brookes, 2005). Studies evaluating the potential effects of fluoxetine exposure on pig (Hroudová and Fišar, 2012) and rat brain mitochondria showed that fluoxetine can decrease Complex I activity and ATP synthesis (Curti et al., 1999). The Ea increase, detected in the present study has also been observed previously in green algae, that accumulated energy reserves as a response to environmental stress such as antibiotic exposure and nutrient deprivation (Cheng and He, 2014; Paes et al., 2016; Aderemi et al., 2018). The energy consumption increase in *P. tricornutum* cells exposed to the highest fluoxetine concentration also triggered a decrease in CEA. According to Verslycke et al. (2004), a decline in CEA indicates a lower net energy budget and, therefore, less energy allocated to vital functions such as growth or cell division (Verslycke et al., 2004). If both main oxygen production and consumption pathways are considered, fluoxetine could possibly affect the diatom-driven oxygenation of marine ecosystems, through a joint process involving a severe depletion of the primary productivity and increased mitochondrial respiration. This will have severe direct cascading impacts on the trophic webs (reduction of the energy input from the primary producers) and reduction of the oxygen availability to the heterotrophs.

The influence of fluoxetine on algae lipid metabolism is mostly unknown but some studies appear to reveal minor impacts (Grzesiuk et al., 2018). Most studies evaluating fluoxetine impacts on lipid metabolism were carried out on vertebrates and seem to agree in that exposure to this drug increases triglycerides abundance, by carboxylesterase expression inhibition and increased fatty acid synthase expression (Feng et al., 2012; Xiong et al., 2014; Pan et al., 2018). Lee et al. (2007) also observed increased arachidonic acid turnover and cytosolic phospholipase A_2_ activity in rats exposed to fluoxetine, which are key components in cell signaling processes in animals, namely in the brain. In photosynthetic microorganisms such as *P. tricornutum* a distinctive fatty acid profile between lipids from thylakoid membranes and extraplastidial lipids, allows to infer which specific lipid classes fluoxetine exposure caused changes, by analyzing the total cellular fatty acid composition (Abida et al., 2015). The increase in C16:2 and C16:3 fatty acids abundance under fluoxetine exposure, which are highly abundant in plastidial galactolipids such as monogalactosyldiacylglycerol (MGDG) and digalactosyldiacylglycerol (DGDG) could possibly indicate an increase of these lipid classes which are involved in FCP complexes stabilization and electron transport mechanisms (Abida et al., 2015). The lipid environment surrounding the photosystems plays an important role in photosystem stabilization and electron modulation at the quinone level (Sakurai et al., 2007; Kern and Guskov, 2011; Mizusawa and Wada, 2012). Although there was no significant variation in LC-PUFA abundance or membrane fluidity parameters, often occurring under stress conditions (Los and Murata, 2004; Feijão et al., 2018), positive correlations between fluoxetine concentration and fatty acid unsaturation parameters such as PUFA, UFA, PUFA/SFA, and DBI, suggest a possible mechanism of fluoxetine favoring fatty acid unsaturation, although this action mechanism is not yet completely understood.

Some of the results presented in this study are in agreement with proposed mechanisms for fluoxetine showing that this molecule can interact with proton efflux pumps and cause algal cell abnormalities (Munoz-Bellido et al., 2000; Brooks et al., 2003). Neuwoehner et al. (2009) reported that fluoxetine does not specifically act on the photosystem II of algae but instead could have a similar mechanism to norflurazon, a specific carotenoid-biosynthesis inhibitor, and therefore target a similar receptor and also impact algal energy budget. However, it cannot be detailed whether the increase in β-carotene abundance was due to a carotenoid biosynthesis blockage or the result of a ROS protection mechanism. To evaluate the possibility of an existent blockage of the carotenoid biosynthesis pathway, real-time PCR could be performed to assess the gene regulation of genes encoding enzymes related to β-carotene synthesis and metabolism. This highly efficient technique could give valuable insights on the regulation of this pathway which also involves other photoprotective pigments like DD and DT that rely on β-carotene as one of their first precursors as previously reported (Kuczynska et al., 2015).

**FIGURE 10:**
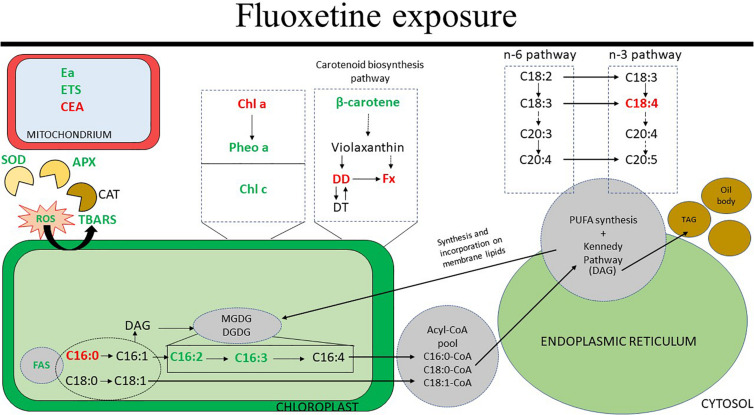
Schematic representation of the physiological effects reported in this study related to fluoxetine exposure of *Phaeodactylum tricornutum* cells for 48 h. The results shown are related to the highest fluoxetine concentration (80 μg L^–1^). Arrows represent simplified (dashed) and direct (full) pathways. Green and red correspond to increased and decreased abundance, respectively. Abbreviations include: APX, ascorbate peroxidase; CAT, catalase; CEA, cellular energy allocation; Chl *a*, chlorophyll *a*; Chl *c*, chlorophyll *c*; DAG, diacylglycerol; DD, diadinoxanthin; DGDG, digalactosyldiacylglycerol; DT, diatoxanthin; Ea, available energy; ETS, mitochondrial electron transport system; FAS, fatty acid synthesis; Fx, fucoxanthin; MGDG, monogalactosyldiacylglycerol; Pheo *a*, pheophytin *a*; PUFA, polyunsaturated fatty acids; ROS, reactive oxygen species; SOD, superoxide dismutase; TAG, triacylglycerol; TBARS, thiobarbituric acid reactive substances. Metabolic pathways addressed are overly simplified versions of the figures published by Kuczynska et al. (2015) and Mühlroth et al. (2013).

The major results of this study are summarized in Figure 10 which highlights the increases in ROS-scavenging mechanisms such as SOD and APX, the variations in the abundance of pigments involved in the FCP complex and variations in chloroplastidial fatty acids.

In ecotoxicological terms, one of the major concerns regarding PPCPs exposure is the lack of efficient biomarkers for certain organisms, specially under the exposure to novel emerging contaminants. In this context, in order to evaluate the two main larger sets of data here presented, bio-optical parameters and fatty acids profile, a CAP analysis was conducted. This procedure allowed disentangling which assessment would prove more suitable to address potential toxicity early-signs of fluoxetine exposure. While bio-optical analyses are non-invasive and can be performed repeatedly, fatty acid analyses require classical biochemical procedures and can only be conducted at specific timepoints with organism destruction. Despite the methodological advantages of bio-optical procedures, in this case, the results could not disentangle the physiological effects of the six exposure groups, specifically we were unable to separate low doses from the control samples. This can be particularly important when addressing matrices with low input of fluoxetine. Nevertheless, bio-optical techniques have the advantage to be non-invasive and produce results in minutes, allowing for repeated-measures during the ecotoxicological trial. On the other hand, in cases of high fluoxetine contamination, this approach proved to be highly reliable, providing an important tool for quick and reliable evaluation of toxicity driven physiological effects from exposure to this pharmaceutical. Regarding fatty acid data, it efficiently separated all the exposure groups with higher classification accuracy, making this a more efficient approach for ecotoxicity assessment using this diatom under fluoxetine exposure at specific endpoints, where culture sacrifice and time are not limiting.

## Conclusion

It is clear from this study that higher concentrations of fluoxetine can affect marine diatoms. Short-term exposure to this pharmaceutical compound caused severe decreases in cell density driven from severe metabolic changes (pigments profile changes, photosynthetic impairment, CEA, and oxidative stress conditions). Although the correspondent photoprotective and antioxidant mechanisms were triggered, increased membrane damage (lipid peroxidation) was still observed. With these metabolic changes, diatom exposure to fluoxetine may have severe implications to marine water oxygenation, carbon harvesting, and EFA production necessary to the upper trophic levels of marine food webs. Not considered in this study were the possible persistence and synergistic effects of fluoxetine with other contaminants commonly found in marine environments, which can further potentiate the negative impacts of pharmaceuticals exposure through synergistic effects (Cabral et al., 2019).

## Data Availability Statement

The raw data supporting the conclusions of this article will be made available by the authors, without undue reservation, to any qualified researcher.

## Author Contributions

BD, VF, and PR-S conceived and designed the experiments. EF, RC, ID, and SN performed the experiments. EF wrote the manuscript. AM, MC, ML, IC, and JM provided the technical and editorial assistance. All authors contributed to the article and approved the submitted version.

## Conflict of Interest

The authors declare that the research was conducted in the absence of any commercial or financial relationships that could be construed as a potential conflict of interest.
